# Towards Personalization of Indoor Air Quality: Review of Sensing Requirements and Field Deployments

**DOI:** 10.3390/s22093444

**Published:** 2022-04-30

**Authors:** Qian Xu, Hui Ci Goh, Ehsan Mousavi, Hamed Nabizadeh Rafsanjani, Zubin Varghese, Yogesh Pandit, Ali Ghahramani

**Affiliations:** 1 Department of the Built Environment, College of Design and Engineering, National University of Singapore, Singapore 119077, Singapore; qianxu@nus.edu.sg (Q.X.); e0202456@u.nus.edu (H.C.G.); 2 Department of Construction Science and Management, Clemson University, Clemson, SC 29634, USA; mousavi@clemson.edu; 3 School of Engineering and Applied Sciences, Western Kentucky University, Bowling Green, KY 42101, USA; hamed.rafsanjani@wku.edu; 4 Trane Technologies PLC Engineering & Technology Centre, Bangalore 560029, India; zubin.varghese@tranetechnologies.com (Z.V.); yogesh.pandit@tranetechnologies.com (Y.P.)

**Keywords:** IAQ, indoor pollutants, ubiquitous IAQ sensors, personalized air quality, sensing standards

## Abstract

As humans spend more time indoors, ensuring acceptable indoor air quality (IAQ) through ubiquitous sensing systems has become a necessity. Although extensive studies have been conducted on the IAQ sensing systems, a holistic review of the performance and deployment of Ubiquitous IAQ Sensing (UIAQS) systems with associated requirements in IAQ sensing standards is still lacking. In this study, we first reviewed IAQ pollutants and other IAQ-related factors and the associated requirements in the prominent IAQ sensing standards. We found that while non-pollutant factors are influential on occupants’ perception of IAQ and their satisfaction, they do not have evaluation metrics in the IAQ standards. Then, we systematically reviewed field studies on UIAQS technologies in the literature. Specific classes of information were recorded and analyzed further. We found that the majority of the UIAQS systems did not meet the requirements of the prominent IAQ sensing standards and identified four primary research gaps. We concluded that a new holistic and personalized approach that incorporates UIAQS measurements and subjective feedback is needed. This study provides valuable insights for researchers and policymakers to better improve UIAQS technologies by developing personalized IAQ sensors and sensing standards.

## 1. Introduction

Indoor Environmental Quality (IEQ) has gained increasing importance since humans spend the majority (up to 90%) of their time indoors [1]. Among factors influencing Indoor Environmental Quality (IEQ), Indoor Air Quality (IAQ) is becoming more prominent as our state of knowledge on the role of air in the spread of pathogens, pollens, and chemical substances advances. These pollutants significantly contribute to Sick Building Syndrome (SBS), which is defined as discomfort or acute health symptoms experienced by occupants in indoor environments [2]. In addition, poor indoor air quality is known to negatively affect occupant productivity [3]. To address the challenges of energy efficiency in buildings and improve occupant comfort, health, and productivity, IAQ standards and codes, such as ASHRAE 62.1, World Health Organization (WHO) 2006, and Singapore Standard SS 554, provide guidelines on IAQ pollutant limits [4].

It is very challenging to monitor all IAQ pollutants with high accuracy and resolution (e.g., accurate measurements at the occupant level) at scale. In addition, other IAQ-related factors, such as odor and humidity that may not directly have adverse impacts on human health, yet may impact on occupants’ satisfaction and comfort, are often not considered via quantifiable metrics in IAQ standards [5]. For instance, although odors may not be directly harmful to human health, they play an essential role in influencing occupants’ mood, alertness, and cognitive performance [6]. Furthermore, studies have shown that humidity can affect the way people perceive IAQ, and it could impact occupants’ emotions [7]. In the absence of sensing methods integrated with IAQ perception, occupants take control of air ventilation systems (e.g., natural ventilation, HVAC systems, and fans) based on their own preferences whenever they are offered the opportunity [8]. Another missing category of airborne hazards is microbes that have become a dominant concern in recent years and more particularly with the emergence of the COVID-19 pandemic. The implications of airborne transmission of COVID-19 causes a paradigm shift in the way that people view indoor air pollutants as particulates, potentially carrying the virus and increasing virus incidence [9].

The recent advancements in IAQ sensing technologies and large-scale deployment of IAQ sensing systems and user interfaces (e.g., kiosks and tablets) have provided the opportunity to collect IAQ data (as defined by IAQ standards) and other related factors at high resolution (e.g., the occupant scale). However, few literature reviews are conducted to holistically study IAQ-related factors, including the intersection of IAQ and sensing technologies. For example, H. Zhang and R. Srinivasan [10] reviewed IAQ sensing standards and current commercially available IAQ sensing technologies. However, the existing literature review studies are not holistic. For instance, while Coulby et al. [11] and Saini et al. [12]’s studies focused on the development of IAQ sensing technologies at the building scale, future studies on the IAQ sensing technologies on a larger scale are needed. More specific information, such as deployment locations and duration, are expected in the future design and deployment of IAQ sensing technologies [13]. In addition, there has been no study to delineate the state-of-the-art field deployment of ubiquitous sensing technologies to evaluate their sensing performance and understand their current practices, shortcomings, and future directions, such as the personalization of IAQ.

To address the above-mentioned IAQ challenges, this study first conducts an in-depth review regarding IAQ standards, as well as Ubiquitous IAQ Sensing (UIAQS) systems that could potentially fulfill the standard requirements at the occupant level and in real-time. UIAQS systems can be defined as sensing systems that can provide real-time and a high spatial and temporal resolution for monitoring indoor air concentration levels [14]. To determine the scope of the review, we considered IAQ pollutants and relevant factors impacting occupant perception of IAQ (e.g., odor and humidity) that are neglected in the existing standards. Then, specific standards regarding the requirements of IAQ sensing systems are thoroughly reviewed. This is followed by a systematic review and a comparison of the UIAQS systems deployed in the field and four major categories of research gaps and future directions. By examining the reviewed articles, this study underlines the importance of integrating personal preferences with UIAQS systems in light of a holistic view of the IAQ. Moreover, this study serves as a useful reference for researchers and practitioners to understand current IAQ standards and personalized sensing technologies, which helps establish personalized IAQ sensing (PIAQS) systems.

The structure of the study is as follows. Section 2 provides a systematic review of primary IAQ-related factors, followed by a critical review of current IAQ sensing standards in Section 3. Section 4 discusses the research design of this study. Section 5 presents the systematic review of UIAQS technologies in the field studies. The research gaps and suggestions are discussed in Section 6. The proposed future research direction–developing personalized IAQ sensing systems is elaborated in Section 7, followed by the conclusions in Section 8.

## 2. Physical Environmental Factors Based on IAQ Standards’ Requirements

In this study, we first provide a short review of the physical environmental factors affecting IAQ based on the IAQ standards requirements, including ASHRAE 62.1, World Health Organization (WHO) 2006, and Singapore Standard SS 554. ASHRAE standards 62.1 is a well-recognized guideline for ventilation system design and indoor air quality, especially where building HAVC systems are used. WHO 2006 air quality standard is widely used in a general indoor environment and the guidelines are now seen as the key source on which the European Commission’s directive on air quality is based [15]. Considering the diversity of climate zones, Singapore Standard SS 554 was also included to represent the IAQ standards adopted in tropical climate regions. The selected physical environmental factors are categorized into four subgroups-IAQ pollutants, odor, humidity, and microbes. Specifically, seven IAQ pollutants, including particulate matter, formaldehyde, carbon dioxide, carbon monoxide, nitrogen dioxide, ozone, and total volatile organic compounds (TVOCs), are selected. Although the temperature is a characteristic of air, we opted not to include it in this review as it is heavily studied within the thermal comfort research domain.

### 2.1. IAQ Requirements on Air Pollutants

- Particulate Matter (PM)

Particulate matter is the most commonly monitored indoor air pollutant, which is defined as a mixture of solid or liquid particles suspended in air [16]. These particles vary in size, shape, and composition (e.g., PM1, PM2.5, and PM10,). Specifically, PM10, PM2.5, and PM1 are the particulate matters with aerodynamic diameters less than 10 μm, 2.5 μm and 1 μm [17]. Particulate matter exposure is harmful and dangerous, as it may lead to a variety of adverse health impacts, including eye, nose, and throat irritation, aggravation of coronary and respiratory disease symptoms, and even premature death in people with heart or lung disease [18]. Specifically, J.M. Logue, P.N. Price, M.H. Sherman, and B.C. Singer (2012) [19] reported that PM2.5 has the significant chronic impact on human health, because they are inhalable and not easily filtered by the human lungs, which may cause the particles to enter the bloodstream. More importantly, PM1 may create greater environmental and health issues among other indoor air pollutants, because these smaller-size particles can be inhaled deeper into the lungs and contaminate human body with toxic and harmful substances than the larger particles [20].

Given the adverse effects caused by particulate matter, various agencies provided guidelines on recommended levels of PM10 and PM2.5. Specifically, ASHRAE 62, Singapore Standard SS 554 and WHO 2006 guidelines provided an identical recommended level of PM10, which is 50 μg/m^3^. WHO 2006 also recommended a limit of PM2.5, which is 25 μg/ m^3^ [21]. However, PM1 has not been addressed by the majority of the IAQ standards.

- Formaldehyde

Following the particulate matter, the second most reported indoor pollutant is formaldehyde (HCHO), which was identified as a Group 1 human carcinogen by the World Health Organization (WHO) in 2004 [22]. In indoor air, formaldehyde can be produced by wood-based products and combustion [23]. As the air-exchange rate may become low in residential dwellings where wood is largely adopted, indoor formaldehyde concentrations tend to be generally higher than outdoors. Guidelines for indoor formaldehyde exposure have been established worldwide. For instance, the WHO set the guideline to 100 μg/ m^3^ in 1987 [24]. Depending on various guidelines, the values of indoor formaldehyde concentration range from 9 μg/m^3^ to 125 μg/m^3^ [25].

- Carbon Dioxide (CO_2_)

CO_2_ is another indoor air pollutant that is closely linked to the ventilation rates and occupancy of a space. While occupant respiration is the primary source of CO_2_, open flame and the use of reinforced concrete could generate significant levels of CO_2_ [26]. Inadequate ventilation or over-occupancy may increase the concentration of CO_2_ indoors. At very low air movements, an occupant may inhale from the CO_2_-rich bubble formed around the breathing zone with significantly higher CO_2_ concentrations compared to the background [27]. Unhealthy levels of CO_2_ concentration indoors may have a negative impact on occupant cognitive functions, productivity, and comfort [28]. Inverse correlations between cognitive performance and concentration levels with CO_2_ have been shown to be the main cause of SBS symptoms [29,30]. A general rule of thumb of the acceptable limit of indoor CO_2_ level is 700 ppm above outdoor [31], which is aligned with the requirement by Singapore Standard SS 554 in 2021 [32]. While based on ASHRAE, the suggested indoor CO_2_ level is 1000 ppm in 2019 [33].

- Carbon Monoxide (CO)

Carbon Monoxide is an odorless and colorless gas that poses potential health hazards to building occupants. Low CO concentrations can cause fatigue in healthy residents and chest pain in people with heart disease [34]. At higher concentrations, it may impair vision and affect coordination and induce headaches, dizziness, confusion or nausea [22]. CO concentration is fatal at very high levels, as carbon monoxide poisoning results in death [35]. The guidelines on the limits of the CO level vary from 6ppm (according to WHO 2006 guidelines) to 9 ppm (according to ASHRAE in 2019 and Singapore Standard SS 554 in 2021) [21,32,33].

- Nitrogen Dioxide (NO_2_)

Nitrogen Dioxide is a by-product of combustion processes. Some common sources of NO2 include stoves, ovens, candles, and mosquito coils. Low-level NO_2_ exposure may increase the risk of respiratory inflammation and infections of building occupants. Extremely high-dose exposure to NO_2_ may result in pulmonary edema and diffuse lung injury [36]. Continued exposure to high NO_2_ levels can contribute to the development of acute or chronic bronchitis [22]. According to WHO 2006 guidelines, the suggested limit of nitrogen dioxide in an indoor environment is 200 μg/m^3^ [21].

- Total Volatile Organic Compounds (TVOCs)

Total volatile organic compounds (TVOCs) are emitted as gases from certain solids or liquids in the building environment. TVOCs can be found in paint, aerosol sprays such as cleansers, disinfectants, air fresheners, and insecticides. They can also be emitted from building materials like office equipment such as photocopiers and printers, correction fluids and carbonless copy paper, and adhesives like furniture glue. TVOCs can cause acute symptoms such as eye, nose, and throat irritation, headaches, loss of coordination, and nausea. Long-term exposure may even bring damage to the liver, kidney, and central nervous system (e.g., tremors and hepatitis) [37]. Singapore Standard SS 554 suggests that the concentration of TVOCs indoors should be less than 3000 ppb in 2021 [32].

- Ozone

Ozone is a common air pollutant worldwide, the levels of which have increased globally in recent decades [38]. Ozone can be generated from a chemical reaction between NO_2_ and TVOCs in exposure to sunlight. The sources of ozone include the emission of chemical solvents, electric utilities, gasoline vapors, and the use of photo-copy devices. It has been widely reported that ozone is associated with respiratory illness, such as cardiovascular mortality [39], stroke [40], and heart failure [41]. People with underlying diseases, children, and the elderly are the highest risk populations for ozone pollutants. Because of the severe consequence of exposure to ozone, the concentrations of ozone in indoor air are regulated by IAQ guidelines. Specifically, the limit of ozone in an indoor environment is 60 ppb based on the guideline of WHO 2006 [22], while the number is 50 ppb according to Singapore Standard SS 554 in 2021 [32]. 

The summary of the potential health effects and various IAQ code of practice for the selected indoor air pollutants is presented in Table 1.

### 2.2. Odor

Compared with the IAQ pollutants listed above, odor may not directly threaten occupant health, but it can potentially affect occupant mood, perception of indoor air freshness, productivity and indicate the presence of mold, which negatively impacts occupant’s health by causing irritation, allergic reactions and other respiratory ailments [6]. One of the most important features of odor is that the perception of odor in human brains can be subjective. The same scent can be labeled as “pleasant” or “unpleasant”, depending on an individual’s preferences. To understand subjective indoor air quality, prior studies have provided occupants questionnaires to collect their perceived IAQ [42]. It has been found that personal factors, such as age and gender, affect occupant perception of good indoor air quality. Although humans might adapt to odor over time, which means a person’s perceived IAQ can be initially unpleasant and changes over long exposure durations, no reviewed IAQ standards included the guidelines on the perceived IAQ for control purposes in the built environment.

### 2.3. Humidity

In addition to common air pollutants, indoor humidity can be categorized as relative humidity (RH) and absolute humidity (AH). Specifically, RH refers to the measure of water vapor present in the air as a percentage of the air vapor capacity (i.e., the amount of water vapor that saturates the air). RH refers to the measure of water vapor relative to the temperature of the air, which is often represented as a measure of humidity indoors. AH refers to the measure of the absolute grains of water vapor in the air, and it is independent of air temperature. Though humidity is not directly an indoor air pollutant, it affects the way occupants perceive IAQ [7]. Current literature supported the claim that RH at an adequate level can positively influence the perception of IAQ [43,44] and sleep quality [45,46]. However, RH less than 30% may lead to the perception of sensory irritants in the air and eye dryness, and RH greater than 70% may cause occupant perception of poor IAQ [47]. Presently, the widely accepted comfortable range of RH is between 30 to 50% [48]. Furthermore, low RH (<30%) can affect human health via two mechanisms. (a) Pathogenic agents’ infectivity and survival have been shown to depend, at least partially, on RH levels [49], and (b) low levels of RH indoors may lead to dry air pathways (i.e., from mouth and nose to the lungs) in the body, which in turn accommodates a likelihood of sickness when one is exposed to infective agents [50].

### 2.4. Microbes

Poor IAQ can lead to a greater virus incidence beyond the multitude of health problems discussed earlier. Previous studies have shown that air pollution particles have the capacity to carry and spread microbes across distances, increasing airborne diseases (such as SARS, Measles, and Chickenpox) [51]. Long-term exposure to indoor air carrying microbes may weaken human respiratory systems and make residents more susceptible to viruses such as COVID-19 [52]. However, given the potential hazards that microbes may cause, acceptable ranges for various microbes have not been determined by the majority of IAQ standards or guidelines.

## 3. Requirements of Prominent IAQ Sensing Standards 

**Figure 1:**
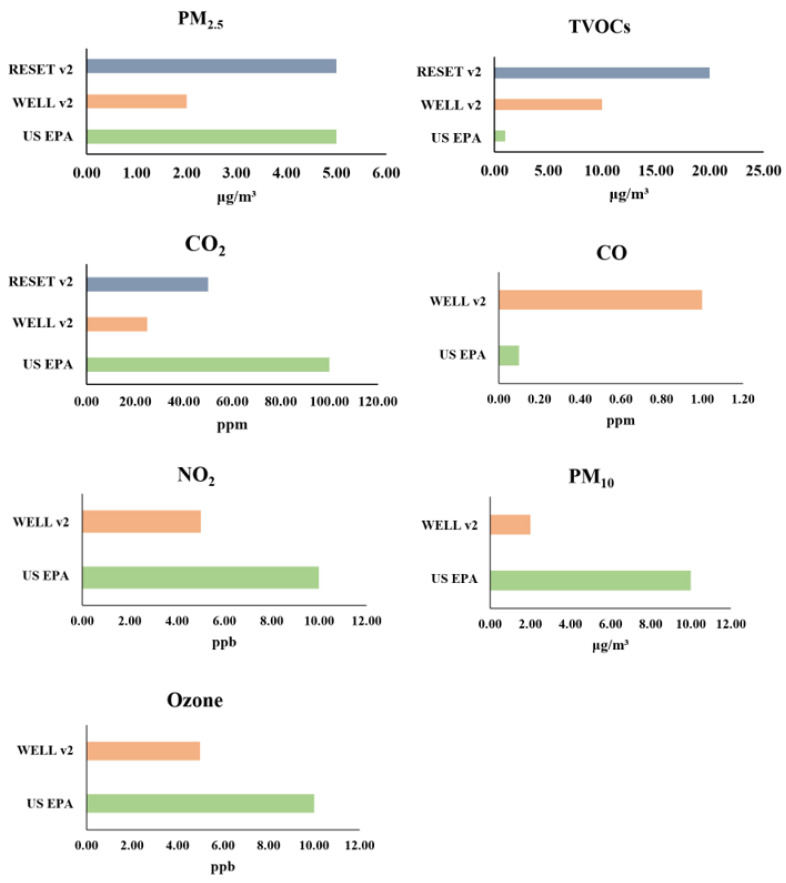
Comparison of the accuracy requirements of three standards on IAQ sensing technologies. Note: As the accuracy requirements are not available in US EPA, minimum output resolution was adopted instead.

With the knowledge of the harmful effects and standards for indoor air pollutants, odor, humidity, and microbes, it is vital to develop guidelines and standards for IAQ sensors in assessing air quality. Many organizations, including the European Union (EU), ASTM international, and the US EPA, are considered as standards assessing air sensor performance. Considering data availability and completion, we compared the accuracy requirements of three commonly recognized specification requirements–US EPA [53], WELL (v2) [54], and RESET (v2) [55], which have been illustrated in Figure 1. As shown in Figure 1, no standardized guidelines for IAQ sensing technologies have been developed. The required air characteristics and the corresponding accuracy requirements vary in different standards. Specifically, US EPA has the most completed requirements on the threshold concentration of indoor air pollutants, while only three air pollutants are included in RESET (v2). Compared with another two standards, WELL (v2) has the strictest accuracy requirements on IAQ sensing technologies except for CO. Such different requirements can be attributed to different organizations or countries who were involved in making the standards. Although the measured pollutants vary in different standards, the air pollutants carbon dioxide, TVOCs, and particulate matter are presented across various guidelines, indicating the prevalence of monitoring these indoor air pollutants.

## 4. Research Methodology

### 4.1. Selection of Academic Publications

**Figure 2:**
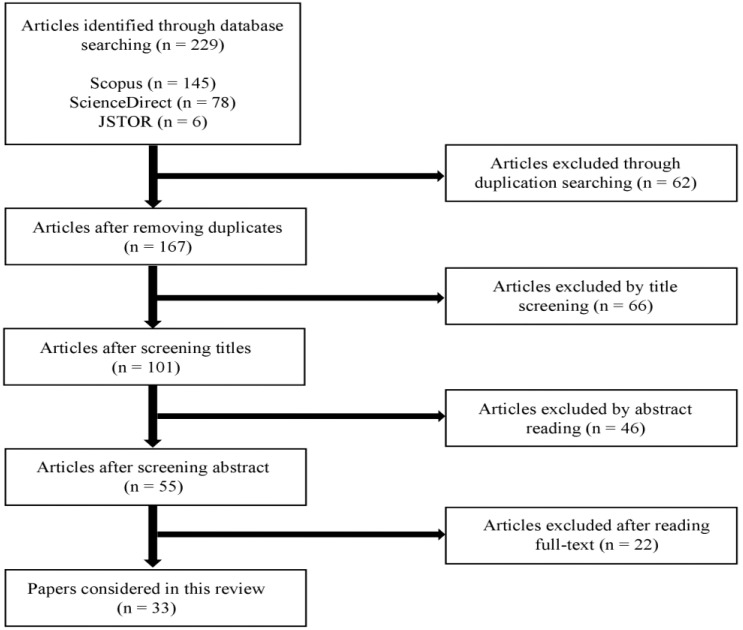
Review procedures.

The definition of Ubiquitous Air Quality Sensing (UIAQS) system has merged from several laboratories for industrial practice in recent years, which refers to the sensing systems that can provide real-time and high spatial and temporal resolution for monitoring indoor air concentration levels [14]. This study follows this definition, which provides the key criteria for the selection of academic articles. The selected academic articles were examined based on a four-step review process, which is illustrated in Figure 2.

In step one, the literature database and search rules are defined. To acquire all potential research articles, the literature search was carried out based on the databases ScienceDirect, Scopus, and JSTOR. As this review mainly focuses on the prior studies related to IAQ sensing systems, other databases, such as PubMed, were not included. The search was limited to the last 12 years of publication (i.e., from 2010 to 2021), as UIAQS technology was not prolific before that. In addition, only peer-reviewed papers published in English were included. In this study, “ubiquitous” and “low-cost” are used interchangeably since the expensive sensors are difficult to be applied on large scales. Thus, the keywords “low-cost or inexpensive, indoor air quality, indoor air pollutant, IAQ sensing technologies” are used to narrow down the selection of relevant articles. The second step is to conduct a preliminary search. The primary emphasis of literature selection involved reading titles and abstracts of each paper to exclude the irrelevant articles. For instance, the articles without critical information, such as measured factors, sensor modules, and deployment location, are excluded in this review. After the preliminary literature selection, literature filtration was conducted through reading the remaining articles to ascertain their relevance. In total, 229 papers were identified, and 33 (13%) papers were rigorously reviewed, while the remaining articles were partially reviewed as supporting records, which were referred to only when needed. The final step involved the categorization of the selected literature based on the categorization rules elaborated on in Section 4.2.

### 4.2. Categorization of UIAQS Technologies

Based on the common features among UIAQS studies, identified IAQ-related factors, and the guidelines for IAQ sensing technologies, this study dissected all selected UIAQS technology-related studies by extracting detailed information on “study location”, “deployment”, “duration”, “measured factors”, “sensor modules”, “measurement range” “accuracy”, “sensor type”, and “occupant information and feedback”. The detailed information of each essential property is presented in Table 2. 

## 5. Findings from Ubiquitous Sensing Technologies in the Field Studies Literature

UIAQS technologies have emerged from different laboratories to practical applications in recent years. Table 3 summarized 33 existing UIAQS studies capable of assessing the concentrations of identified IAQ-related factors. 

**Figure 3:**
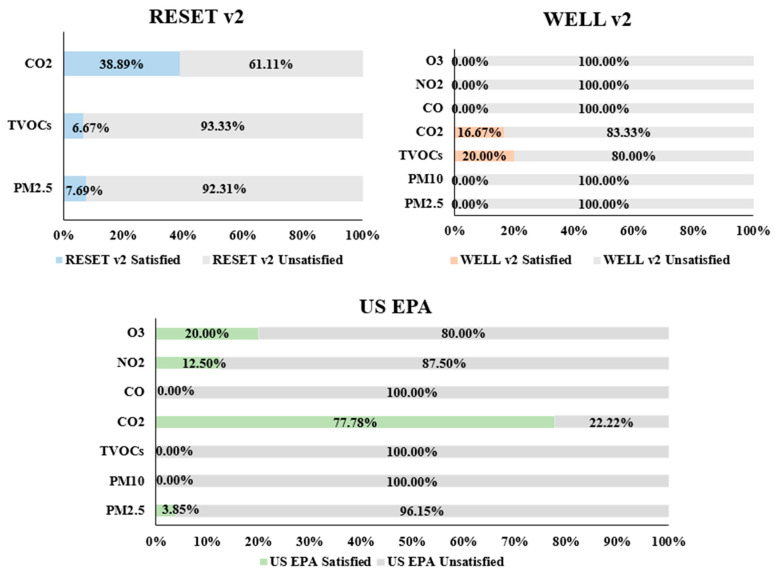
Sensor modules of various IAQ pollutants and sensing requirements. Note: CO2 and CO_2_, PM2.5 and PM _2.5_, PM10 and PM_10_, NO2 and NO_2_, and O3 and O_3_ are used interchangeably.

As shown in Table 3a, when assessing IAQ-related factors, different studies adopted various sensing modules, which may have different measurement ranges and accuracy rates. Given the three IAQ sensing standards reviewed in Section 3, Figure 3 illustrates the percentage of the reviewed sensor modules (with various tested air pollutants) that satisfied or unsatisfied the requirements of sensing standards US EPA, WELL v2, and RESET v2. The majority of the deployed IAQ sensors failed to fully meet the requirement of current commonly adopted IAQ sensing standards. Specifically, none of the sensing modules detecting PM_10_ satisfy any of the selected IAQ sensing standards. Sensors measuring CO_2_ have the highest percentage of fulfilling any one of the three IAQ sensing standards. Over 75% of the CO_2_ sensors meet the requirements of US EPA, while the percentage of CO_2_ sensors that satisfy the requirements of RESET v2 drops to 38.89%. Only 16.67% of the CO_2_ sensors meet the requirements of WELL v2.

The occupant information and feedback during the field studies are shown in Table 3b. 6 out of 33 studies provided relevant information. The information and feedback include occupant demographic information (e.g., living conditions), socio-economic status (e.g., highest qualifications), and their attitudes towards IAQ. The online scoring system, questionnaires, and interviews are the commonly adopted methods used to collect the relevant data.

**Figure 4:**
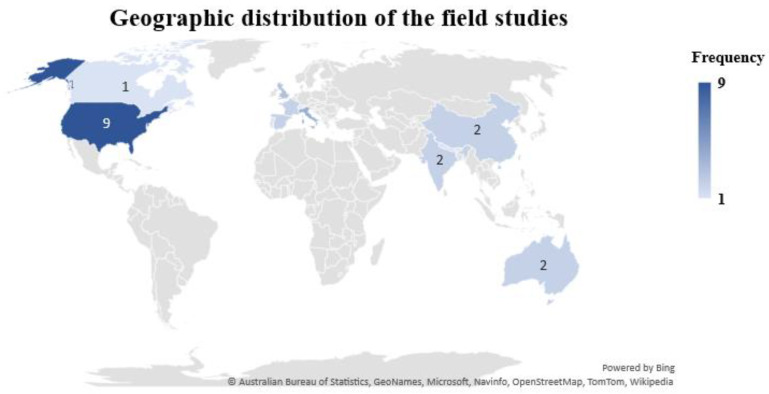
Geographic distribution of the reviewed studies in UIAQS technologies.

The studies analyzed and included in this review are reported from different locations of the world. Out of the 33 studies, 9 (27.27%) field studies were carried out in the United States, while the rest of the studies are scattered across different countries or regions, including China, Australia, and the European Union. Possible reasons that explain why more studies have been done in the United States is that the UIAQS technology is more advanced and acknowledged in the United States. Figure 4 represents the distribution of included studies throughout the world.

**Figure 5:**
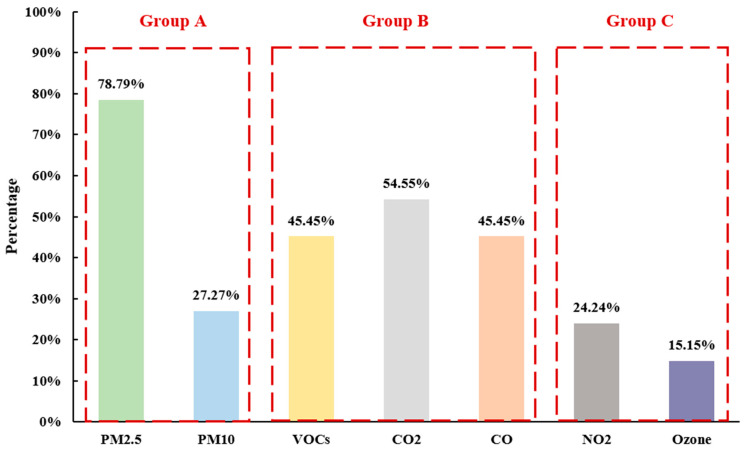
Distribution of the tested IAQ factors. **Note:** CO2 and CO_2_, PM2.5 and PM_2.5_, PM10 and PM_10_, and NO2 and NO_2_ are used interchangeably.

Figure 5 illustrates the main IAQ sensor types reviewed in this study. Seven types of IAQ sensors can be categorized into three groups. Specifically, sensors that take up more than two-third of the total analyzed IAQ sensing systems are in Group A. The most prevalent type of sensors-PM_2.5_ sensors are the only sensor type that can be included in Group A, which account for 78.79% (26 out of 33 articles) of the total IAQ sensing systems. Group B includes the types of IAQ sensors that contribute to more than one-third and less than two-thirds of the analyzed IAQ sensing systems. In particular, sensors detecting TVOCs, CO_2_, and CO are in Group B, which hold 45.45%, 54.55%, and 45.45%, respectively. The last group—Group C—are those types of sensor that take up less than one-third of all the IAQ sensing systems, which include NO_2_ and O_3_ sensors. 

## 6. Research Gaps and Suggestions

This section covers a detailed discussion of four major research limitations of current UIAQS technology deployment and proposes potential solutions for further studies.

### 6.1. Research Gaps of Current UIAQS Technologies

- Sensing IAQ factors

It can be seen from Figure 5 that the distribution of UIAQ sensing factors is uneven. PM_2.5_ sensors take the leading position that more than 75% of the selected studies have PM_2.5_ sensors, while the sensing systems testing ozone concentration only account for 15.15% of the whole. None of the reviewed UIAQ sensing systems have a holistic measurement metric of odor, which is essential for IAQ-related factors that directly impact occupant perception of IAQ in the built environments. The lack of innovative IAQ sensor systems capturing some of the pertinent air pollutants, such as PM_1_, and sub-micron particles, may bring the issue of cross-sensitivity of sensors and the inability to monitor the holistic IAQ.

- Lack of standardization

The second research gap is that the calibration of the UIAQ sensors has not been standardized, which decreases the accuracy of the measurement. The lack of standardization also indicates that the sensor performance is hard to compare with that of the reference sensors [76,81,82].

- Applicability of UIAQS systems in extreme climates

From the geographic perspective, a vast number of studies are conducted in the temperate climate zone. The applicability of the UIAQS technologies to different regions has not been fully evaluated. Therefore, the implications of UIAQS systems drawn from the literature may not be applicable to buildings that are located in different climate zones. For instance, the humidity level in tropical areas is different from that of temperate zones, which has significant effects on the performance of UIAQS systems. Specifically, previous studies demonstrated a positive relationship between RH and sensor output (e.g., [88,89]).

- Inadequate study duration

It has been found that the majority of field studies were conducted for relatively short durations, ranging from a couple of days to a few months. The evaluation of UIAQ sensors with adequate periods (i.e., over one year) is lacking in the literature. However, given the potential seasonal effects on sensor performance, research on the performance of UIAQS systems in field studies over a full year is definitely scarce.

### 6.2. Future Research

Based on the research limitations from the review, this study provides four suggestions for future directions of the development of UIAQS technologies. First of all, as suggested by prior studies (e.g., [56,61,66]), sensors detecting less pertinent IAQ pollutants and other IAQ-related factors, such as odor and humidity, are needed to be integrated into personalized UIAQS systems. As occupants’ perception of odors may vary from each other, no sensors have been deployed to clarify odors in a shared space. However, personalized UIAQS systems with the integration of occupant feedback of IAQ through questionnaires or interviews via interfaces have great potential to provide holistic IAQ measurements, including odors and other IAQ-related factors. In addition, with the emergence of COVID-19, it is crucial to explore the possibility of virus detection based on a long-range wireless sensor network that includes UIAQ sensors and a smart platform with analytics for airborne virus management [75]. Secondly, we suggest that researchers shall consider standardizing the UIAQS system calibration processes, as one of the major challenges identified is the lack of standardized and in-device calibration [57,68]. Thirdly, improving the diversification of the location of deployed UIAQ sensors is highly encouraged. The evaluation of UIAQ sensing performance can be undertaken in various locations across different regions of various climates. It is important to ensure that the performance of UIAQ sensors is consistent across different geographic locations. Last but not least, the evaluation of UIAQ sensors with adequate periods (i.e., over one year) is lacking in the literature [90]. Given the potential seasonal effects on sensor performance, the durations of IAQ testing studies are suggested to be extended for at least one year to account for seasonal effects [90]. The detailed development of integrating UIAQS systems and controls with human perception is elaborated in Section 7.

## 7. Integrating Air Quality Sensing and Controls with Human Perceptions

Relatively high airspeeds (generated by ventilation systems) and humidity impact both IAQ and thermal comfort at the same time. In most buildings nowadays, however, we have relatively low air speeds and an approximately fixed humidity, and the interrelationships become less prominent. In this case, thermal comfort is primarily impacted by air temperature [91], while IAQ is dominated by air pollutants and odor. To optimize IAQ, thermal comfort, and building energy simultaneously, we may need to adjust airspeeds, humidity rates and temperature accordingly to occupants’ instant needs, and that requires more sophisticated and comprehensive control systems, which require utilizing personalized sensors and actuators [92,93,94,95].

**Figure 6:**
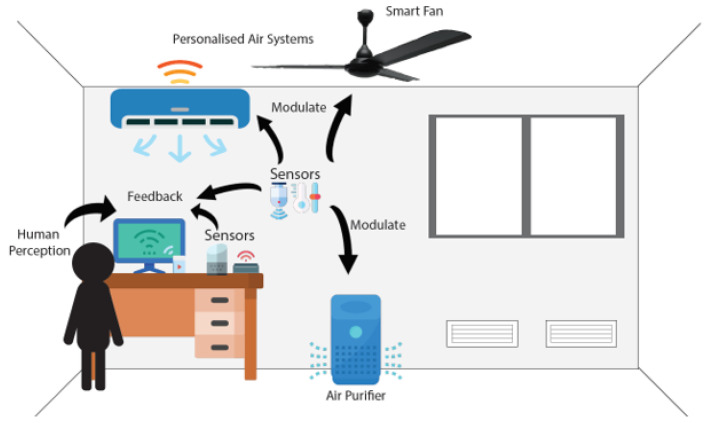
Personalized Indoor Air Quality Sensing (PIAQ) System.

Following the future directions analyzed above, this section discusses how UIAQS technologies can holistically improve occupant health and thermal comfort spatiotemporally, as well as building energy efficiency through personalization of the IAQ. Personalization in this context refers to the development of IAQ sensing systems that can satisfy occupant requirements at the individual level. As illustrated in Figure 6, the PIAQS system requires the integration of occupant perception, ubiquitous IAQ sensors, and IAQ controls. Specifically, non-perceptible indoor air pollutants could be measured by physical sensors and occupant perception and feedback of IAQ can be captured via a user interface. The occupant feedback, in turn, may help adjust IAQ controls and ventilation.

To modify the micro-climate and provide a preferable indoor environment for occupants, IAQ controls and ventilation are essential in the PIAQS systems. Based on different types of UIAQ technologies, various solutions of IAQ controls can be adopted in personalized UIAQS systems [96]. For the stationary UIAQS systems, mechanical filtration [97], biofiltration [98], UV-C photolysis [99], UV-PCO [99], and air ionizers [100] are the most commonly used IAQ control methods. As for the mobile UIAQS technologies, except for biofiltration, other solutions applicable to the stationary UIAQ systems are acceptable. Specifically, mechanical filtration refers to the process of forcing air through fibrous media. Botanical biofiltration uses a mixture of plant species to remove targeted pollutants in the ambient air. UV-C Photolysis has frequently been used to inactivate microbes, such as SARS-CoV-2-virus, in recent years. UV-PCO is a process that seeks to remove TVOCs and NO from the air using oxidation, which can be incorporated into the ducting of HVAC systems or air purifiers [101]. Air ionizers make use of the process of ionization to clean the air, which can also be integrated into ventilation ducts of an HVAC system [102]. It is suggested that ion generators can remove small particles from the indoor air and improve occupant perception of IAQ. However, the application of ion generators in IAQ control is still controversial, as they are not effective at removing gases, odor, and large particles [103].

Ventilation is another essential component to improve IAQ in the built environment. One of the purposes of ventilation is to introduce fresh air into space by diluting indoor pollutant concentration levels [104]. Appropriate and adequate ventilation can help reduce the spread of viruses and microbiological contaminants at the individual level [105]. Personalized ventilation can adopt natural ventilation and mechanical ventilation. Natural ventilation uses wind and buoyancy to provide fresh air and to potentially increase air exchange rates [106,107]. However, due to the unpredictability of wind, natural ventilation is suggested to be combined with mechanical ventilation, which usually adopts fan-driven systems to provide clean air to occupants [108]. In extreme climates with very low or high humidity (e.g., deserts or tropical climates), natural ventilation may cause humidity-related challenges.

## 8. Conclusions

With the recent advancements in the IAQ sensing technologies, UIAQ systems are increasingly becoming instrumental in ensuring an individual’s health and comfort in the built environments. This study first critically reviewed the physical environmental factors that affect IAQ and the primary IAQ standards. It was discovered that the non-pollutant IAQ-related factors, such as odor, humidity, and microbes, are not properly addressed in the IAQ standards. However, these factors may have a diverse impact on occupant health, productivity, and their perception of indoor air quality. We then thoroughly reviewed the IAQ sensing standards, including US EPA, WELL v2, and RESET v2. It has been found that the guidelines for IAQ-sensing technologies are not standardized, and the requirements for different IAQ-related factors are varied. In addition, the sensing requirements on non-pollutant IAQ-related factors are exclusive in the guidelines for IAQ sensing technologies as well.

Accordingly, we systematically selected studies on ubiquitous sensing technologies deployed in the field studies from the literature. Of the relevant journal articles, 33 out of 229 distributed in 13 countries were selected for deeper analysis. We then extracted and analyzed several information items such as “study location”, “deployment”, “duration”, “measured factors”, “sensor modules”, “measurement range” “accuracy”, “sensor type”, and “occupant information and feedback”. We found that the majority of the UIAQ sensors could not satisfy the prominent IAQ sensing standards. Among the sensors, CO_2_ sensors have the highest percentage of 77.78% and 38.89% meeting the requirements of US EPA and RESET v2, respectively. Then, based on the UIAQS technologies reviewed in the literature, we identified four major research gaps in the applicability of current UIAQS technologies, including (1) sensing IAQ factors; (2) accuracy shortcoming; (3) applicability of UIAQS systems in extreme climates; and (4) inadequate study duration. Accordingly, four specific suggestions for future research directions are proposed to further improve UIAQS systems in an indoor environment, which include integrating personalization into UIAQS systems.

This study provides a critical reference for both researchers and practitioners studying IAQ sensing technologies in an indoor environment. The proposed “PIAQS” system provides a potential solution to provide personalized experience to the occupants. Researchers can utilize the concept of “personalization” in the context of the indoor environment and provide customized IAQ sensing and control strategies by collecting occupant perception and feedback. Policymakers are suggested to consider non-pollutant IAQ-related factors into the IAQ sensing standards to better guide the development of IAQ sensing technologies. Future research may focus on addressing the challenges of PIAQS technologies, thereby extending and enriching its application in practice.

## Figures and Tables

**Table 1:** The potential health effects and recommended levels of indoor air pollutants.

Air Pollutant	Potential Health Effects	IAQ Code of Practice		
		ASHRAE	Singapore Standard SS 554	WHO 2006 Guidelines
Carbon Dioxide	Headache/Fatigue/Nausea/Dizziness	1000 ppm	700 ppm above outdoor	N/A
Carbon Monoxide	Fatigue/Impaired vision/Reduced brain function/Nausea/Headaches/Dizziness/Flu-like symptoms/Fatal	9 ppm	9 ppm	6 ppm
Formaldehyde	Asthma/microvascular leakage/cancer	0.1 ppm (office)	0.1 ppm	0.1 ppm
		0.04 ppm (home)		
Nitrogen Dioxide	Eye, nose, throat irritation/Acute or chronic bronchitis/Respiratory infections	N/A	N/A	200 μg/m^3^
Ozone	Respiratory illness, such as cardiovascular mortality	N/A	50 ppb	60 ppb
TVOCs	Eye, nose and throat irritation/Nausea/Headaches, loss of coordination/Damage to liver, kidney, and central nervous system/Skin irritation	N/A	3000 ppb	N/A
Particulate Matter	Eye, nose, and throat irritation/Aggravation of respiratory tract related ailments	50 μg/m^3^ (PM_10_PM_10_)	50 μg/m^3^ (PM_10_PM_10_)	50 μg/m^3^ (PM_10_PM_10_)
				25 μg/m^3^ (PM_2.5_PM_2.5_)

**Table 2:** Categorization of the selected UIAQS technology-related studies.

Category	Description
Study location	Study location revealed the country, the location where the study was conducted (e.g., kitchen, living room, etc.). The rationale behind identifying the country is to grasp a better sense of the country’s climate.
Deployment	The deployment column recorded the specific locations where the sensors were installed (e.g., on the desk, near fans, etc.). This information helps identify the various tested factors and the sensors’ suitability for different scenarios.
Duration	Duration revealed the time length of the studies, such as seven days or one year.
Measured factors	The measured factors recorded the type of indoor environmental factors that affect IAQ.
Sensor Modules	The sensor modules column kept track of the sensor brand and model name.
Measurement range	The measurement range documented the measuring range of each environmental factor
Accuracy	The accuracy information was extracted from the specifications of the sensors, which helps in evaluating the suitability of the sensors when deployed in various settings.
Sensor type	Sensor type indicated whether this UIAQ sensor is a stationary or a mobile sensor.
Occupant information and feedback	Occupant information and feedback took a record of the contextual information and feedback from the occupants via online feedback, questionnaires, and interviews.

**Table 3:** (a). Air quality sensing systems characteristics, deployment, and study information. (b). Summary of occupant information and feedback collected in the literature.

(a)								
Study	Study Location	Deployment	Duration	Measured Factors	Sensor Modules	Measurement Range	Accuracy	Sensor Type
[1]	Laboratory France	Sensor 1: in front of green wall, 0.7 m above floor Sensor 2 and 3: Near fans Sensor 4: in a corner of room	-	CO	Figaro TGS2442	30–1000 ppm	-	S
				CO_2_	Figaro TGS4161	350–10,000 ppm	±20%	
				TVOCs	Figaro TGS 2602	1–30 ppm	-	
				O_3_	E2v MICS 2610	10–10,000 ppb		
				NO_2_	E2v MICS 2714	0.05–5 ppm		
[56]	Residential Spain and India	Spain: 1 m from an indoor fireplace and 0.6 m above the ground India: main living area at least 1 m above the ground	Spain: 5 days India: 7 days	CO	EL-USB-CO	3–1000 ppm	±7 ppm	S
				${\mathrm{PM}}_{2.5}$	HAPEx Nano	$8–150\;\mathrm{mg}/m^{3}$	-	
[57]	Laboratory USA	-	-	CO_2_	MG811	350–10,000 ppm	-	S
				TVOCs	TGS2602	1–30 ppm		
				CO	MQ7	20–2000 ppm		
				O_3_	MQ131	10–1000 ppb		
[58]	Residential Canada	1 m above a drawer at the center of a room	7 days	${\mathrm{PM}}_{2.5}$	Shinyei Kaisha PPD42-60	$0–200\;\mu g/m^{3}$	±20 μg/m^3^ or 10%	S
				CO_2_	ELT Sensor T-110-3V	400 to 10,000 ppm	±50 ppm or 3%	
				CO	Figaro Engineering TGS5342	0–1000 ppm	±10 ppm	
				TVOCs	Cambridge CMOS CC881B	0–1000 ppb	±10 ppb or 5%	
				O_3_	SGX Sensortech MICS-2714	0–1000 ppb	±10 ppb or 5%	
				NO_2_	SGX Sensortech MICS-2714	0–1000 ppb	±10 ppb or 5%	
[59]	Residential China	1 m above the ground	4 days	${\mathrm{PM}}_{2.5}$	PMS 3003	$0–500\;\mu g/m^{3}$	$100–500\;\mu g/m^{3}:$ $\pm 10\%\;0–100\;\mu g/m^{3}:$ $\pm 10\;\mu g/m^{3}$	S
[60]	Residential USA	1 to 5 m from stove or furnace	2 months	CO	Alphasense COB4	0–45 ppm	±10 ppm	S
[61]	Laboratory USA	-	-	CO_2_	Telaire T6713	0–5000 ppm	±30 ppm +3%	S
				TVOCs	MiCS-5524	1–1000 ppm	-	
				CO	MiCS-5524	1–1000 ppm		
				${\mathrm{PM}}_{2.5}$	Itead DSM501A	-		
				${\mathrm{PM}}_{10}$	Itead DSM501A	-		
				Formaldehyde	WSP2110	1–50 ppm		
				NO_2_	MiCS-2714	0.05–10 ppm		
[62]	Office UK	On a shelf (1.65 m high) above a computer desk	1 month	TVOCs	CCS-811	0–1187 ppb	-	S
				CO_2_	MH-Z19B	0–2000 ppm		
				${\mathrm{PM}}_{2.5}$	PMSA003i	$0–500\;\mu g/m^{3}$		
				Humidity	BME280	0–100%		
[63]	Laboratory Switzerland	On the table at 0.75 m above the ground	1 h	CO_2_	Sensirion SCD40	0–40000 ppm	±50 ppm ±5%	S
					CO2meter K30	0–10,000 ppm	±30 ppm ±3%	
				${\mathrm{PM}}_{2.5}\;$ $\mathrm{and}\;{\mathrm{PM}}_{10}$	NovaFitness SDS018	$0–1000\;\mu g/m^{3}$	max of ±15% & ± 10 μg/m^3^	
[64]	Residential USA	On a table in the main living space	7 days	CO	Alphasense COB4	0–1000 ppm	±10 ppm	S
				CO_2_	Netatmo	0–5000 ppm	±50 ppm, ±5%	
				NO	Alphasense NOB4	0–20 ppm	±10 ppb	
				NO_2_	Alphasense NO2B43F	0–20 ppm	±50 ppb	
				${\mathrm{PM}}_{2.5}$	Alphasense OPC-N2	0.38–17 μm	-	
[65]	Residential, campus and church USA	Living room, Classroom, and Church	-	TVOCs	TGS2602	1–30 ppm	-	S
				NO_2_	GSNT11	0–200 ppm	±10%	
				CO	TGS5042	0–10,000 ppm	-	
				SO_2_	SO2-AF	400–700 ppm		
				${\mathrm{PM}}_{2.5}$	GP2Y1010AUF	-		
				${\mathrm{PM}}_{10}$	GP2Y1010AUF			
[66]	Laboratory USA	-	10 days	CO_2_	CO2Meter K-30	0–10,000 ppm	±30 ppm ± 3%	S
				${\mathrm{PM}}_{2.5}$	GP2Y1010AU0F	$0–0.5\;\mathrm{mg}/m^{3}$	-	
				TVOCs	CO2Meter IAQ-2000	350–2000 ppm		
[67]	-	Test chamber	-	CO_2_	SenseAir K30	0–10,000 ppm	±30 ppm +3%	S
				TVOCs	AMS CCS811	0–1200 ppb	-	
					SGX Sensortech MICS-VZ89TE	0–2000		
					Bosch BME680	-		
					Bosch BME688			
[68]	Residential USA	Near the edge of a stable surface in the center of the living room	2 months	${\mathrm{PM}}_{2.5}$	AQE2	-	-	S
					BlueAir Aware	$1–500\;\mathrm{ug}/m^{3}$		
					Foobot	$0–1300\;µg/m^{3}$	±4 µg or ±20%	
					Speck (DSM501A)	-	-	
[69]	Laboratory Portugal	-	-	CO	MICS-6814	1–1000 ppm	-	S
				NO_2_	MICS-6815	0.05–10 ppm		
				C_2_H_5_OH	MICS-6816	10–500 ppm		
				H_2_	MICS-6817	1–1000 ppm		
				NH_3_	MICS-6818	1–500 ppm		
				CH_4_	MICS-6819	>1000 ppm		
				C_3_H_8_	MICS-6820	>1000 ppm		
				C_4_H_10_	MICS-6821	>1000 ppm		
[70]	Residential and commercial Australia	Personal monitor carried by people	1 week	${\mathrm{PM}}_{2.5}$	Shinyei PPD60PV-T2	-	-	M
[71]	Residential UK	0.9 m above a drawer	4 days	${\mathrm{PM}}_{2.5}$	SHARP GP2Y1010AU0F	$0–1300\;µg/m^{3}$	$\pm 4\;µg/m^{3}$	S
				TVOCs	AMS iAQ-CORE-C	125–1000 ppb	±1.0 ppm	
				CO_2_	AMS iAQ-CORE-C	400–600 ppm	±1.0 ppm	
[72]	Hospital Italy and Spain	2.5 m and 1 m above the ground	Multiple months	${\mathrm{PM}}_{2.5}$	Syhitech DSM501A	-	-	S
					Model 3321 Aerodynamic Particle Sizer			
					SPK202			
					SPK201			
				TVOCs	Corvus	0–50 ppm	±5 ppb	
				CO_2_	ZyAura	0–3000 ppm	±75 ppm or ±5%	
[73]	Commercial Australia	In Office buildings	-	CO	TSI Q-Trak 7575	500–2000 ppm	±3% or ±50 ppm	S
				CO_2_	Fieldpiece SCM4	0–15 ppm	±5% or ±1 ppm	
				${\mathrm{PM}}_{2.5}$	TSI DustTrak II 8532	$0–0.1\;\mu g/m^{3}$	-	
				${\mathrm{PM}}_{10}$	TSI DustTrak II 8532	$0–0.1\;\mu g/m^{3}$		
				Formaldehyde	HalTech HFX205	0–500 ppb		
[74]	Residential India	Integrated in the kitchen (areas around the cookstove) and room	Multiple days	${\mathrm{PM}}_{2.5}$	GP2Y1010AU0F	-	-	S
[75]	Hospital	1.2 m above the ground at the center of each room	24 h	TVOCs	Sensirion SVM30	0–60,000 ppb	1.3%	S
					Renesas ZMOD4410	0–1,000,000 ppb	±25%	
				CO_2_	Sensirion SVM30	400–60,000 ppm	1.3%	
					Renesas ZMOD4410	400–5000 ppm	±25%	
				${\mathrm{PM}}_{2.5}$	Sensirion SPS30	$1–1000\;µg/m^{3}$	$0–1000\;µg/m^{3}:$ $\pm 10\;µg/m^{3}$	
				${\mathrm{PM}}_{10}$	Sensirion SPS30	$1–1000\;µg/m^{3}$	$0–100\;µg/m^{3}:$ $\pm 25\;µg/m^{3}$ $100–1000\;µg/m^{3}:$ ± 25%	
[76]	Residential Nepal	1.5 m height and about 1 m from the edge of the main stove and ≥1 m from any doors or other openings in the walls	13 months	${\mathrm{PM}}_{2.5}$	UCB-PATS	$25–25,000\;µg/m^{3}$	-	S
[77]	Laboratory France	1.4 m above the ground on the wall behind occupants	8 months	CO_2_	NDIR	0–5000 ppm	50 ppm	S
					IQ 610	0–10,000 ppm	50 ppm	
				C_6_H_6_ andCO	MQ135	10–1000 ppm	±5%	
				Formaldehyde	MS1100	0–1000 ppm	±3%	
				${\mathrm{PM}}_{2.5}$	GP2Y1010AU0F	$0–500\;\mathrm{mg}/m^{3}$	$0.1\;\mathrm{mg}/m^{3}$	
[78]	Residential Scotland	1 m above the ground in living area, away from possible PM sources	2 months	${\mathrm{PM}}_{2.5}$	Dylos DC1700	-	-	S
[79]	Laboratory Italy	On an evaluation board	5 months	CO	TGS-5042	0–10,000 ppm	-	S
					MICS-4514-CO	0–1000 ppm		
				CO_2_	Gascard NG	0–2000/3000/5000 ppm	±2% of range ±<0.015% of range per mbar	
					S-100	0–2000/3000/5000/10,000 ppm	±30 ppm ±5%	
				NO_2_	NO2B4	0–20 ppm	-	
					NO2_3E50	0.3–50 ppm		
					MICS-2710	0.05–5 ppm		
					MICS-4514-NO2	0.05–10 ppm		
					CairClip NO2	0–250 ppb		
				O_3_	O3B4	0–5 ppm		
					O3_3E1F	0.1–1 ppm		
				NO	NO_3E100	0–100/200 ppm	45 nA/ ppm ± 15 nA/ ppm	
[80]	Laboratory Italy	-	-	NO	Citytech NO_3E100	0–1000 ppm	45 ± 15 nA/ppm	S
				CO	Figaro TGS-5042	0–10,000 ppm	1.2–2.4 nA/ppm	
					e2V MICS-4514	0–1000 ppm	−0.0051(Rs/R0) /ppm	
				CO_2_	Edinburgh Gascard	0–1000 ppm	1 V/100 ppm	
					ELT S-100H	0–5000 ppm	1 V/1000 ppm	
[81]	Residential and commercial Scotland	Integrated in a backpack worn by subjects	2 months	${\mathrm{PM}}_{2.5}$	Dylos 1700	-	-	S
[82]	Laboratory UK	On a 0.75–1.2 m height desk in front of occupant’s work areas	-	Humidity	SHT31	0–100%	±2% RH	S
				${\mathrm{PM}}_{2.5}$ $/{\mathrm{PM}}_{10}$	HPMA115S0	$0–1000\;\mu g/m^{3}$	±15%	
				TVOC1	CCS811	0–1200 ppb	-	
				TVOC2	iAQ-CoreC	125–600 ppb		
				TVOC3	MiCS-VZ-89TE	0–1000 ppb		
				CO_2_	T6713	0–5000 ppm	±25 ppm	
				CO	LLC110-102	0–1000 ppm	±2 ppm	
[83]	Office Spain	Inside an office with no human presence	-	${\mathrm{PM}}_{2.5}$	HM-3301	$1–1000\;\mu g/m^{3}$	-	S
				${\mathrm{PM}}_{10}$	HM-3301	$1–1000\;\mu g/m^{3}$		
[84]	Laboratory	On or adjacent to a wire shelving unit in the central area, several meters from source activities	-	${\mathrm{PM}}_{2.5}\;$ $\mathrm{and}\;{\mathrm{PM}}_{10}$	Air Quality Egg 2018 (AQE)	$0–500\;\mu g/m^{3}$	$0–100\;\mu g/m^{3}$ : ±10 μg/m^3^ 100–500 μg/m^3^: ±10%	S
					IQAir AirVisual Pro (AVP)	$0–1798\;\mu g/m^{3}$	-	
					Awair 2nd Edition (AW2)	-	$0–100\;\mu g/m^{3}$: $\pm 15\;\mu g/m^{3}\;100–1000\;\mu g/m^{3}$: ±15%	
					Kaiterra Laser Egg 2 (LE2)	$0–500\;\mu g/m^{3}$	$0–100\;\mu g/m^{3}$: $\pm 10\;\mu g/m^{3}$ 100–500 μg/m^3^: ±10%	
					PurpleAir Indoor (PAI)	$0–500\;\mu g/m^{3}$	$0–100\;\mu g/m^{3}$: $\pm 10\;\mu g/m^{3}$ $100–500\;\mu g/m^{3}$: ±10%	
					Ikair (IKA)	$0–1000\;\mu g/m^{3}$	$0–100\;\mu g/m^{3}$: $\pm 10\;\mu g/m^{3}$ $100–1000\;\mu g/m^{3}$: ±10%	
[85]	Residential USA	On a table in the living room, 3 m from the main entrance and 10 m from the kitchen	12 months	${\mathrm{PM}}_{2.5}$	AirVisual Pro	-	±8%	S
[86]	Laboratory and residential USA	1.1 m above the ground, 0.5 m away from a wall, and at least a 1.5 m away from any corner	7 days	${\mathrm{PM}}_{2.5}\;$ $\mathrm{and}\;{\mathrm{PM}}_{10}$	Nova Fitness SDS011	$0.0–999.9\;µg\;/m^{3}$	$15\%\;\mathrm{or}\;\pm 10\;µg/m^{3}$	S
				NO_2_	SPEC Sensors DGS-NO2 968-043	0–10 ppm	±15%	
				SO_2_	SPEC Sensors DGS-SO2 968-038	0–20 ppm	±15%	
				CO_2_	CO2Meter K-30	0–5000 ppm	±30%	
				CO	SPEC Sensors DGS-CO 968-034	0–1000 ppm	±15%	
				O_3_	SPEC Sensors DGS-O3 968-042	0–5 ppm	±15%	
				TVOCs	Ohmetech.io uThing:VOC™	0–500 IAQ index	±15%	
[87]	Residential China	Sensor 1: 1.2 m above the ground in adjacent room Sensor 2: in a shoulder brace 0.15 m away from cook’s nose	1 month and 5 days	${\mathrm{PM}}_{2.5}$	AM510	-	-	S
(**b**)								
**Study**		**Occupant Information and Feedback**						
[59]		A brief survey about each home, its residents, and general behavior patterns, such as home parameters, cooking days, other potential indoor PM sources.						
[70]		Questionnaires on participants’ age and gender, socio-economic status (highest qualification) and characteristics of the indoor and outdoor environments during weekdays and weekend, such as age for their residence, ventilation conditions at home and work, type of cooktop at home and commuting preferences.						
[76]		Questionnaires about three groups of variables: (1) Variables for the day of monitoring, such as type of primary and secondary stoves used in the house, unusual stove use pattern during the HAP monitoring period, weather conditions during the monitoring period, ventilation in the kitchen (e.g., open doors and windows) and other smoke exposure sources, such as number of smokers in the house, use of incense or mosquito coils. (2) Fixed variables, such as kitchen size or the presence of roads within 100 m. (3) Variables describing usual practices, such as types of non-electric lamp used when power is unavailable, and type of space heating used during the winter. Interviews about participants’ caregivers or parents about their occupations and household characteristics, such as construction materials.						
[78]		Questionnaires on occupant attitudes towards smoking and interest in having a device placed at home to measure air quality.						
[81]		Interviews and online questionnaires on personal data, such as individual’s living conditions, the household size and accommodation details, building and neighborhood characteristics and other contextual factors.						
[82]		Feedback through an online IEQ scoring system.						

Note: (1) Unreported: “-”. (2) Sensor Type: S (Stationary); M (Mobile).

## Data Availability

Not applicable.
